# Evaluation of bioluminescent imaging for noninvasive monitoring of colorectal cancer progression in the liver and its response to immunogene therapy

**DOI:** 10.1186/1476-4598-8-2

**Published:** 2009-01-07

**Authors:** Maider Zabala, Pilar Alzuguren, Carolina Benavides, Julien Crettaz, Gloria Gonzalez-Aseguinolaza, Carlos Ortiz de Solorzano, Manuela Gonzalez-Aparicio, Maria Gabriela Kramer, Jesus Prieto, Ruben Hernandez-Alcoceba

**Affiliations:** 1 Division of Gene Therapy and Hepatology. CIMA, University of Navarra. Foundation for Applied Medical Research. Av. Pio XII. Pamplona, Spain; 2 Morphology and Imaging Unit. CIMA, University of Navarra. Foundation for Applied Medical Research. Av. Pio XII. Pamplona, Spain; 3 CIBERehd. University Clinic of Navarra. Pamplona, Spain; 4 Institute for Stem Cell Biology and Regenerative Medicine, University of Stanford, 1050 Arastradero Road, Palo Alto, CA, USA; 5 Peter MacCallum Cancer Research Institute, Cancer Immunology Program, St Andrews Place, East Melbourne, 3001 Australia

## Abstract

**Background:**

Bioluminescent imaging (BLI) is based on the detection of light emitted by living cells expressing a luciferase gene. Stable transfection of luciferase in cancer cells and their inoculation into permissive animals allows the noninvasive monitorization of tumor progression inside internal organs. We have applied this technology for the development of a murine model of colorectal cancer involving the liver, with the aim of improving the pre-clinical evaluation of new anticancer therapies.

**Results:**

A murine colon cancer cell line stably transfected with the luciferase gene (MC38Luc1) retains tumorigenicity in immunocompetent C57BL/6 animals. Intrahepatic inoculation of MC38Luc1 causes progressive liver infiltration that can be monitored by BLI. Compared with ultrasonography (US), BLI is more sensitive, but accurate estimation of tumor mass is impaired in advanced stages. We applied BLI to evaluate the efficacy of an immunogene therapy approach based on the liver-specific expression of the proinflammatory cytokine interleukin-12 (IL-12). Individualized quantification of light emission was able to determine the extent and duration of antitumor responses and to predict long-term disease-free survival.

**Conclusion:**

We show that BLI is a rapid, convenient and safe technique for the individual monitorization of tumor progression in the liver. Evaluation of experimental treatments with complex mechanisms of action such as immunotherapy is possible using this technology.

## Background

The liver is the most frequent site for metastases from colorectal cancer. Approximately 10–25% of colon cancer patients present one or multiple liver metastases at the time of diagnose [1]. At least in 30% of these cases the liver is the only organ affected, apart from the tumor in the gastrointestinal tract. Moreover, recurrence after surgical removal of the primary lesion occurs mainly in the liver, with a 20–25% rate of metachronous liver metastases. Potentially curative resection of hepatic tumors is not feasible in more than 75% of the cases due to large size, elevated number and/or unfavourable localization of lesions, or poor liver function. Nonsurgical approaches including systemic chemotherapy and regional treatments are the only options for these patients. Local control is often achieved and these techniques are rapidly improving [2,3], but a significant increase in long-term survival is not guaranteed. Therefore, hepatic metastases from colon cancer are frequently observed in the clinic and they are the most frequent cause of death in these patients. Advances in the management of this disease will probably require the combination of standard care and new therapies that are still in the experimental stage.

Immunotherapy is one of these alternatives [4]. Systemic or local administration of vectors driving expression of immunostimulatory cytokines such as interleukin-12 (IL-12) has demonstrated potent antitumor effects in pre-clinical studies [5-8]. However, further optimization of this approach is required, and improvement in animal models is needed so that research in this area can generate more clinically relevant results [9,10]. In a previous study [11], we described a High-Capacity (*gutless*) adenoviral vector carrying a liver-specific inducible system for the expression of murine IL-12 (GL-Ad/RUmIL-12). Intravenous administration of this vector eliminated intrahepatic colon cancer in a murine model when intense production of IL-12 was induced at early stages of the disease. If more restrictive conditions are used (larger tumors and lower dose of vector that leads to moderate IL-12 concentration) the antitumor response was heterogeneous (manuscript in preparation), as observed with many other experimental approaches [12].

In these cases, a more detailed characterization of the partial responses would be desirable, and longitudinal monitoring of individual subjects could identify transient antitumor effects. Implantation of certain colon cancer cell lines in the liver of syngeneic mice constitutes one kind of intrahepatic cancer model [13]. Although each model has its own limitations, progressive growth and extra hepatic dissemination of these tumors often leads to the death of the animal, recapitulating some aspects of the natural history found in humans. However, monitoring progression in these internal tumors by direct measurement requires repeated laparotomy or large groups of animals to be sacrificed at different time points, thus precluding an individualized follow-up. Different noninvasive imaging techniques have been developed to overcome these limitations. Some of them such as ultrasonography (US) [14], computerized tomography (CT) [15], positron emission tomography (PET) [16], single photon emission computed tomography (SPECT) [17] and magnetic resonance imaging (MRI) [18,19] are adaptations of clinical imaging devices to the use in small animals. Others such as fluorescence imaging (FLI) [20] and bioluminescent imaging (BLI) [21,22] have been specifically developed for the *in vivo *monitoring of gene expression in experimental animals, mostly rodents.

Bioluminescence of cells is based on a chemical reaction catalyzed by the luciferase enzyme in which a substrate (D-luciferin) is converted into an excited oxyluciferin intermediate in the presence of Oxygen, Magnesium and ATP [23]. When oxyluciferin returns to its relaxed state, it emits a photon in the visible wavelength range. The most common source for luciferase is the firefly *Photinus pyralis*. Since no luciferase expression is found in mammalian cells and there is no need for external light excitation, this method of cell labelling has a very low background. The intensity of light is proportional to the amount of luciferase expressed in each individual cell, and the number of cells in which the gene has been transferred. In addition, actual light detectors have a very high dynamic range. Therefore, luciferase-based assays have been widely used to study changes in gene expression intensity *in vitro *and *in vivo*. For tumor imaging, a luciferase expressing gene is transferred to cancer cells and stable clones are implanted into permissive hosts. These can be syngeneic animals [24-27], but in the case of human cancer cell lines, immunodeficient mice or rats are required [28-32]. A cooled charge-coupled device (CCD) camera attached to a light-tight chamber is then used to detect the intensity and location of light emission. The high sensitivity of BLI has been extensively demonstrated in different tumor models, including colon cancer [30,33]. However, stability of luciferase expression and maintenance of a good correlation between light emission and tumor burden needs further investigation, especially if orthotopic (intrahepatic) immunocompetent models are used [26,27]. The influence of anatomical barriers and the fact that luciferase is a foreign gene could interfere with this correlation. Therefore, in this study we have stably transfected MC38 murine colon cancer cells with a plasmid carrying luciferase gene and have monitored the evolution of bioluminescence and tumor volume after subcutaneous or intrahepatic inoculation of cells in C57BL/6 mice. We found a direct correlation between these two parameters in both cases, although higher dispersion was observed at late stages of the experiment. Long-term maintenance of the genetically modified cells and stability of luciferase expression were observed.

In addition, we provide evidence showing that this model is suitable for evaluation of individual responses to immunotherapy. Using BLI monitoring, we were able to identify subsets of animals that experienced transient reduction in tumor progression, and to clearly distinguish this group from others having no effect, or long-term complete responses.

## Results and discussion

### Characterization of MC38 colon cancer cells stably transfected with the luciferase gene

**Figure 1:**
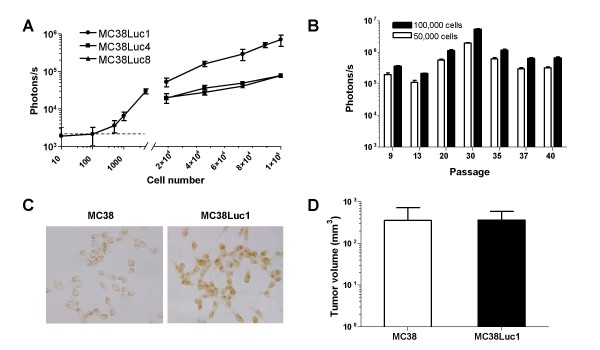
**Characterization of the MC38Luc1 cell line**. A.-Three independent MC38Luc clones were seeded in 96-well plates at different cell densities, and incubated with the luciferase substrate D-luciferin. Light emission from living cells in each well was immediately quantified using a CCD camera. The horizontal discontinuous line indicates the background light emission of an empty well. B. – The same procedure was used to quantify light emission from clone 1 (MC38Luc1) after maintenance in vitro for at least 40 passages. C. – Expression of luciferase was detected in MC38Luc1 cells at passage 30 by immunocytochemistry (right panel). Left panel corresponds to the parental MC38 cells. Magnification 400×. D. – MC38Luc1 or the parental MC38 cells (10^6 ^per mouse) were injected subcutaneously in the right hind flank of C57BL/6 mice (n = 10) to compare tumorigenicity. Tumor volumes achieved 3 weeks after cell implantation are shown. Error bars represent standard deviations.

MC38 cells were transfected with a plasmid carrying luciferase under the control of the CMV promoter, and 10 different clones were isolated after antibiotic selection. As expected, clones varied in their specific luciferase activity (measured by standard luminometer -data not shown-). In figure 1A we represent light emission in living cells corresponding to 3 clones after incubation of increasing number of cells with the substrate D-luciferin. Clone number 1 (MC38Luc1) was chosen for further characterization because it presented the highest luciferase activity. We determined that at least 500 MC38Luc1 cells are required to obtain a luminescence significantly higher than background (3,590 +/- 1,275 versus 1,900 +/- 1,300 photons/s, respectively). Intense light emission was maintained for at least 40 passages *in vitro*, as shown in figure 1B. A transient increase in the specific luciferase activity (photons/s/cell) was observed until passage 30, with further stabilization (figure 1B). This rules out the possibility that the CMV promoter is inhibited over time in the MC38Luc1 clone. Luciferase expression was analyzed by immunocytochemistry (figure 1C) in order to verify that the transgene is expressed in all individual cells at late passages. The lack of negative cells supports the absence of promoter silencing.

In order to verify that the MC38Luc1 cells retained the tumorigenic properties of the parental cells, we inoculated 10^6 ^MC38 or MC38Luc1 cells subcutaneously in two groups of C57BL/6 mice. We could not detect any difference in tumor progression between both groups. In figure 1D, we represent the average tumor volume 3 weeks after inoculation of cells, determined by direct calliper measurement. Therefore, integration of the luciferase expression cassette in the genome of the MC38Luc1 clone did not change their ability to form tumors *in vivo*. In a previous report by an independent group [33], MC38 cells transduced with retroviruses encoding luciferase or GFP showed moderate differences in tumor progression, although both cell lines gave rise to tumors that grew progressively for at least 20 days. The size of tumors observed in our experiments (367 mm^3 ^after 21 days) coincides remarkably with the clone described by Choy et al [33]. This and other studies in syngeneic animals clearly demonstrate that tumor cells expressing luciferase are not selectively eliminated by the immune system.

### Comparison of US and BLI for noninvasive monitorization of liver metastases

**Figure 2:**
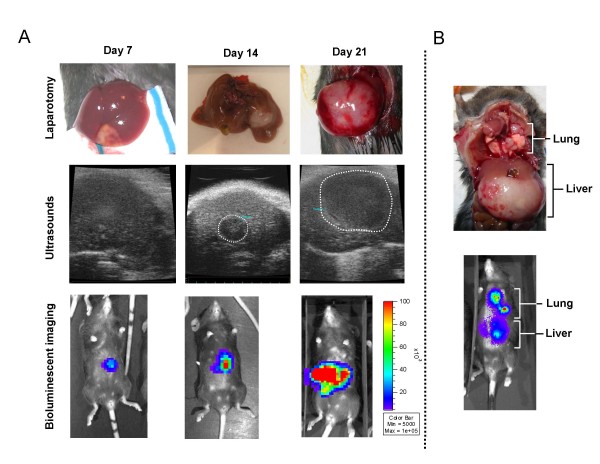
**Monitorization of hepatic tumors by BLI and US**. A.-MC38Luc1 cells were injected in the liver of C57BL/6 mice (n = 20), and tumor progression was evaluated weekly by either BLI (bottom pictures), US (middle pictures) or direct tumor measurement through laparotomy or necropsy (upper pictures). Margins of tumors are indicated with a dotted line in the US images. B.-Example of lung metastases secondary to the hepatic lesions 4 weeks after cell implantation in the liver. Detection by BLI (bottom picture) and confirmation after necropsy (upper picture).

**Figure 3:**
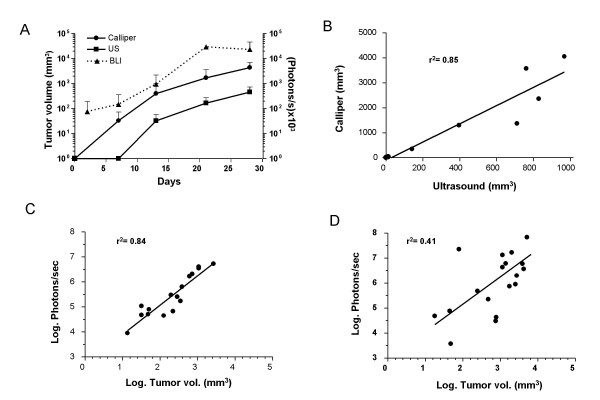
**Correlation between BLI, US and direct tumor volume determination in hepatic tumors**. MC38Luc1 cells (5 × 10^5^) were injected in the liver of C57BL/6 mice (n = 20), and tumor progression was evaluated weekly by either BLI, US or direct tumor measurement through laparotomy or necropsy. A – Tumor volume determined by direct measurement (calliper, black circles) or US (black squares) over time. Volumes are indicated in the left Y axis. Light emission quantified by BLI is represented as a dotted line and the scale corresponds to (photons/s) × 10^3 ^in the right Y axis. Error bars represent standard deviations. B.-Correlation between tumor volumes determined by US (X axis) and direct measurement after necropsy (calliper, Y axis) one month after cell implantation. C-D – Correlation between light emission and direct tumor measurement 2 weeks (C) or 3 weeks (D) after cell implantation (log_10 _conversion of values).

We inoculated 5 × 10^5 ^MC38Luc1 cells in the liver of C57BL/6 mice and monitored tumor progression by BLI or US. Subsets of animals in the group were laparotomized or sacrificed at one week intervals for direct calliper measurement of tumor diameters. In figure 2A, we show representative examples of colorectal metastases identified by the three methods at different stages of the disease. Both BLI and US were able to detect the presence and localization of lesions, and to assess their progression over time. US was less sensitive and could not detect the small tumors rising one week after cell injection. However, this technique provided more anatomical information about tumor spread and involvement of other organs. As expected, BLI had lower spatial resolution, but it was able to indicate the approximate location and size of tumors in most of the cases, apart from the light emission quantification. In fact, in those few animals that survived more than 5 weeks, lung metastases usually occurred and could be detected by BLI (figure 2B). Finally, BLI is faster (at least 10 mice can be analyzed at a time in less than 20 minutes) and requires less technical training than US. In order to validate the noninvasive imaging of liver metastases, we analyzed the correlation between BLI, US and direct tumor measurement. In figure 3A we show the progression of the average tumor volume determined by laparotomy or US, as well as the luciferase activity (light emission in photons/s). From this comparison, we can see that tumor progression can be monitored using both BLI and US. In agreement with previous results from other groups [22], BLI is the most sensitive technique, because tumor cells can be detected as early as 2 days after implantation, before they form macroscopic tumors. The maximum background light emission detected in a non-tumor bearing animal was 10,000 photons/s, approximately 10 times less than the average emission of MC38Luc1-injected animals. US started to detect hepatic lesion 2 weeks later. In our experience, tumor diameters determined by US are smaller than those obtained by direct calliper measurement and therefore the estimated volume of tumors is reduced. We believe this is due to the restrictive criteria used to define tumor margins in the US and does not affect the validity of the technique. In fact, correlation between tumor volumes calculated by both methods is good (figure 3B).

### Limitations of BLI in the advanced stage of tumors

**Figure 4:**
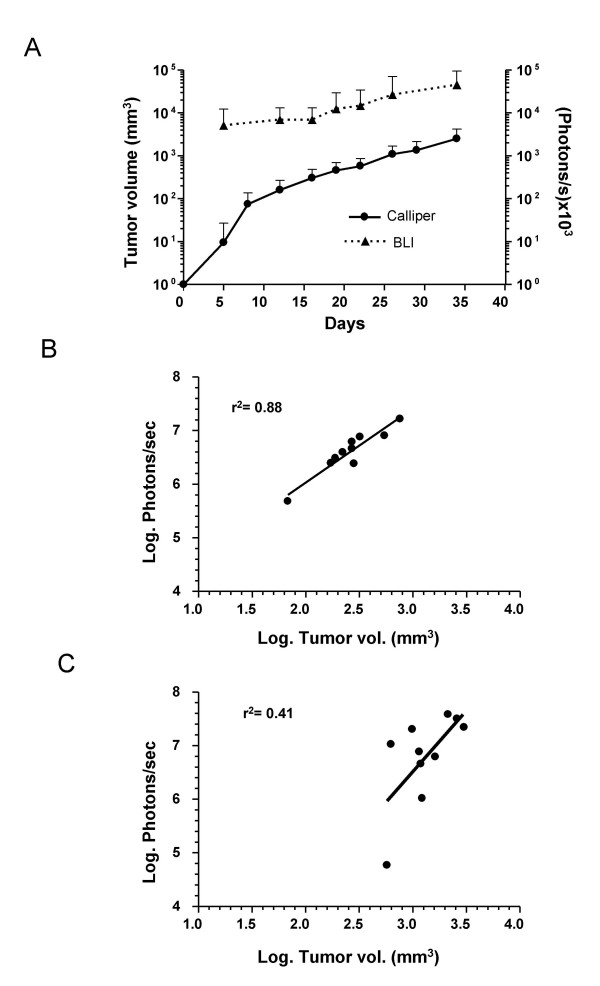
**Correlation between BLI and direct tumor volume determination in subcutaneous tumors**. MC38Luc1 cells (10^6^) were injected subcutaneously in the right hind flank of C57BL/6 mice (n = 14), and tumor progression was evaluated weekly by either BLI or direct measurement. A – Monitorization of tumor volume by direct measurement (calliper, black circles) corresponds to the left Y axis. Light emission quantified by BLI is represented as a dotted line and the scale corresponds to photons/s × 10^3 ^in the right Y axis. Error bars represent standard deviations. C-D – Correlation between light emission and direct tumor measurement 2 weeks (C) or 4 weeks (D) after cell implantation (log_10 _conversion of values).

Good correlation between BLI and direct tumor measurement was also observed during the first 3 weeks of the experiment. In figure 3C, we represent the values for individual animals at day 13 after cell implantation. Later on, when tumors became large enough to be detected by physical examination, linear correlation was partially lost (figure 3D). In general, this is due to a plateau in the luciferase activity of tumors (figure 3A), which does not cope with tumor growth in the later stages of the disease. To investigate if this is specific for intrahepatic tumors, we inoculated MC38Luc1 cells subcutaneously and analyzed the same parameters. In figure 4A we show the average tumor volume and light emission over time. As expected in superficial tumors, high bioluminescence was observed at early time points, and there was a progressive increase following tumor growth. In terms of bioluminescence/volume correlation, it was optimal during the first 3 weeks (figure 4B), and then the values started to be more dispersed (figure 4C). Therefore, the same trend is observed in subcutaneous and hepatic tumors, although the partial loss of correlation is more obvious in the hepatic lesions, probably because they grow faster. This suggests that BLI is less reliable to assess tumor burden in advanced stages of the disease, when tumors approach their maximum size. Nevertheless, it can be useful to determine non-invasively a criteria for animal sacrifice before they reach the undesirable situation of extremely large lesions, thus contributing to the refinement of intrahepatic tumor models. In this case, it seems that light emission higher than 2.5 × 10^7 ^photons/s could be considered a reasonable limit.

**Figure 5:**
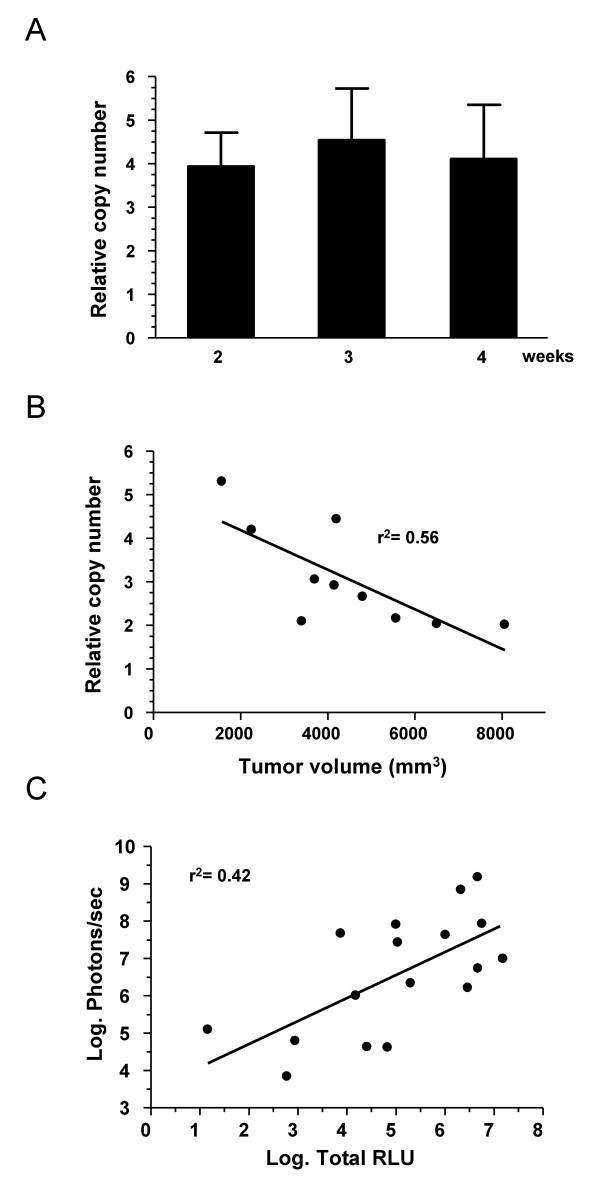
**Analysis of luciferase stability and function *in vivo***. A. – Intrahepatic MC38Luc1 tumors (n = 10) were excised at different times after cell implantation (2, 3 and 4 weeks), and the relative copy number of the plasmid pCDNA3-luc was determined by qPCR. The values correspond to the number of plasmid copies divided by the copies of the endogenous albumin gene. Error bars represent standard deviations. B. – Correlation between the relative pCDNA3-luc copy number in advanced tumors (4 weeks after implantation) and tumor volume. C. – Correlation between *in vivo *light emission of advanced tumors (in photons/sec.) and luciferase activity (in RLUs) obtained in solution from tumor extracts (log_10 _conversion of values).

It is not clear at this moment if the limitations of BLI in large tumors can be considered a general characteristic of this technique. The observation periods described in the literature are heterogeneous, and the time to reach the "advanced stage" differs in each tumor model. Nevertheless, attenuation of luciferase signal has been previously described in another colorectal cancer liver metastasis model developed in Balb/c mice [27]. A partial loss of correlation between BLI and physical measurement was found in large tumors starting on day 20 after cell inoculation, which is similar to our observations. In this case the main reason was the quenching effect of the ascitic fluid, which is usually abundant in advanced CT26 tumors growing in the liver. Accumulation of large volumes of hemorrhagic ascites was less frequent in our intrahepatic MC38Luc1 tumors. In addition, this factor cannot play a role in the subcutaneous tumors described here. In a different study, a marked inhibition of bioluminescence was found in a bladder cancer model, starting 2 weeks after cell implantation [34]. The authors demonstrated a reduction in light emission from necrotic and hemorrhagic areas of the tumors. In addition, these areas can contribute to the quenching effect of surrounding tissues. If the proportion of these inactive fractions of the tumor increases over time and is more prevalent in large tumors, this is a feasible explanation for the loss of correlation between bioluminescence and tumor volume. In our model, histological analysis of tumors revealed variable regions of necrosis and haemorrhage. However, when we studied individual tumors, we could not establish a direct correlation between the abundance of these areas and the decline of light emission (data not shown). Therefore, other factors may play a role in this phenomenon. We have verified by quantitative PCR that there is no decrease in the frequency of luciferase plasmids integrated in the tumors at different time points. As shown in figure 5A, we found an average of approximately 4 copies of plasmid for every copy of the endogenous albumin gene (which means 2 copies per genome) even in the very advanced stage (day 28). This indicates that the MC38Luc1 cells do not lose the luciferase gene over time *in vivo*, and the proportion of these cells in the tumor mass is maintained on average. Interestingly, the ratio of copies/genome is not randomly distributed among tumors at day 28 (figure 5B). We observed that larger tumors tend to present less copies of luciferase plasmid per genome, suggesting that stromal cells (fibroblasts, endothelial cells, etc.), which do not contribute to the luciferase expression are more abundant in these tumors. These luciferase-inactive components of the tumors, together with areas of necrosis and haemorrhage are mixed in variable proportions and impair an accurate determination of tumor volume by BLI. Therefore, in advanced stages of our tumor model, the functional information obtained by BLI prevails over the morphological criteria.

Hypoxia has been recently proposed as a potential inhibitor of the luciferase reaction [35]. Experiments performed *in vitro *demonstrated that intense hypoxia (0,2% pO_2_) can reduce ATP levels in cells and impair bioluminescence. Differences in the extension and intensity of hypoxic areas could contribute to the lack of correlation between light emission and tumor volume. To investigate this possibility, we obtained lysates from advanced tumors and performed *in vitro *luciferase reactions. Under these circumstances, luciferase activity (measured in Relative Luciferase Units, RLUs in a standard luminometer) only depends on the amount of functional enzyme, because all other components of the reaction, including ATP, are contained in the assay buffer. Therefore, if lack of ATP is limiting light emission in some of the MC38Luc1 tumors *in vivo*, luciferase activity should be restored *in vitro*. However, we did not observe any sample in which low photon emission *in vivo *was accompanied by an unexpectedly high RLU value *in vitro *(figure 5C). This suggests that hypoxia is not interfering with BLI in the MC38Luc1 model. However, the correlation between both parameters was modest (r^2 ^= 0.42) due to considerable dispersion of values. Therefore, we cannot definitely rule out the possibility that availability of ATP *in vivo *is affecting the accuracy of BLI.

### Validity of BLI to monitor the efficacy of immunotherapy

**Figure 6:**
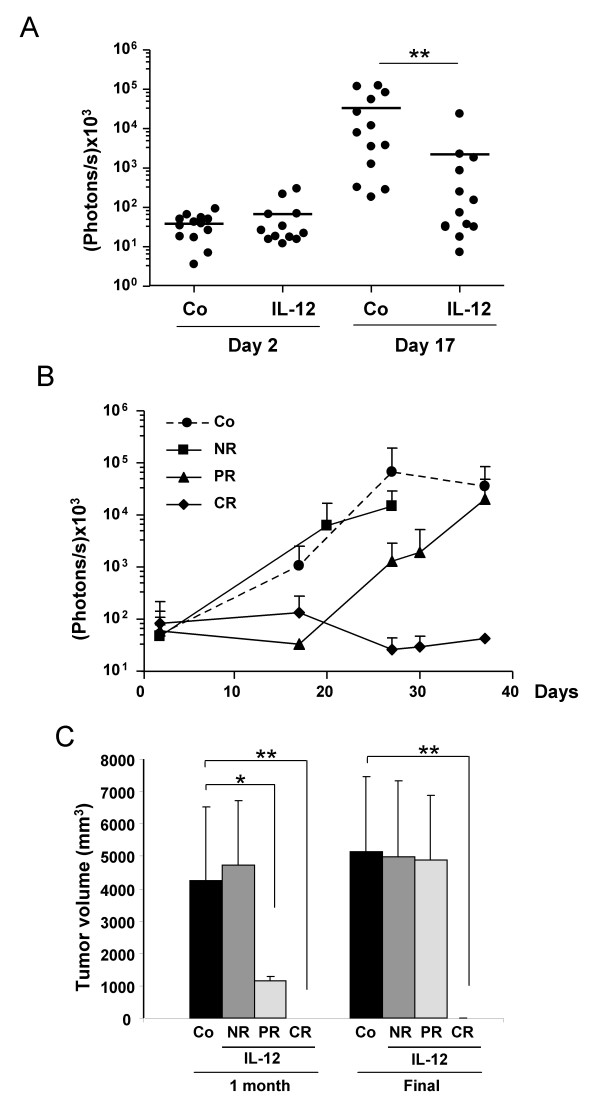
**Antitumor effect of IL-12 monitored by BLI**. MC38Luc1 cells (5 × 10^5^) were injected in the liver of C57BL/6 mice (n = 26). After verification of cell engraftment, the vector GL-Ad/RUmIL-12 was injected intravenously at 2.5 × 10^8 ^iu/mouse in half of the mice, and IL-12 expression was activated by mifepristone one week later. A. – Quantification of light emission before virus injection (day 2) and 7 days after initiation of IL-12 expression (day 17) in control (Co) and treated groups (IL-12). B. – Monitoring of tumor progression by BLI. Control group is represented as a dotted line. Three subgroups were defined among treated animals: non-responders (NR), mice with partial response (PR) and complete response (CR). Differences were statistically significant (p < 0.01) between Co and PR groups during days 20 to 30. Light emission from the CR group remained significantly lower until the end of the experiment. C. – Average tumor volume of the different groups one month after cell implantation, and at the time of sacrifice or spontaneous death. No evidence of tumor was observed in the CR group. Error bars represent standard deviations. * p < 0.05; ** p < 0.01.

The ability of BLI to evaluate the efficacy of conventional [24] or experimental [30,31] treatments has been demonstrated in different tumor models, but little is known about immunotherapy approaches in syngeneic mice. We have previously described a potent antitumor effect of the high-capacity adenoviral vector GL-Ad/RUmIL-12 on intrahepatic MC38-derived tumors [11]. A liver-specific, Mifepristone-inducible expression system allows controlled expression of IL-12. The vector was administered one week before cell implantation, and activation of IL-12 expression started 5 days later. Under these circumstances, tumor eradication was observed in most of the animals when high doses of IL-12 were achieved. Now we aim to evaluate the efficacy of GL-Ad/RUmIL-12 in a more restrictive setting. BLI was used with the objective of determining the onset and duration of the antitumor response in each animal. In the actual protocol, tumor cells (MC38Luc1) were implanted before administration of the virus. Initial BLI performed 48 hours after cell inoculation allowed us to verify the localization of cells in the liver area and the homogeneity of experimental groups in terms of light emission (figure 6A). The next day, a moderate dose of GL-Ad/RUmIL-12 vector (2.5 × 10^8 ^iu) was administered intravenously, and induction of IL-12 expression started one week later. Therefore, tumors had progressed for 10 days before the treatment effectively started. The induction regime consisted on 10 daily injections of 250 μg/kg Mifepristone intraperitoneally, as previously described [11]. The average concentration of IL-12 in serum was 27 ng/ml after the first mifepristone administration, which is consistent with the dose of virus used. Bioluminescence quantification detected significant differences between the control and treated groups as early as 7 days after initiation of IL-12 expression (day 17 after cell inoculation, figure 6A). More importantly, subsequent BLI monitorization (figure 6B) allowed us to distinguish 3 subsets of animals inside the treatment group: non-responders (NR), in which light emission was similar to the control group; mice showing a partial response (PR) consisting in a transient inhibition of luciferase activity; and finally others in which the signal was completely and permanently abolished (CR). All animals that survived long enough were laparotomized 4 weeks after initiation of the experiment in order to determine the size of liver tumors by direct calliper measurement. As shown in figure 6C, stratification of mice according to bioluminescence was in accordance with the changes observed in tumor volume. In the PR group, a significant reduction was observed during the 4^th ^week of the experiment, but the tumors progressed and all animals finally died during the next 4 weeks because of their hepatic lesions. In contrast, mice in the CR group remained tumor-free and were negative for luciferase activity for the entire duration of the experiment (more than 6 months).

## Conclusion

We have validated an immunocompetent model of hepatic tumors that allows the noninvasive monitorization of tumor progression using BLI. A good correlation between functional imaging and tumor volume was observed until mice reached an advanced stage of the disease. This model is suitable to characterize transient and long-term antitumor responses obtained with new therapeutic approaches such as immunotherapy.

## Methods

### Cell lines

The MC38 murine colon carcinoma cell line was derived from 3-methylcholanthrene-treated C57BL/6 mice [36]. It was grown in Dulbecco's modified Eagle medium (DMEM) supplemented with 10% heat-inactivated fetal bovine serum (FBS), 100 μg/ml streptomycin and 100 μg/ml penicillin (all from of Gibco, Invitrogen) and 2 mM glutamine (Cambrex). The MC38Luc1 clone was maintained in the same media supplemented with 400 μg/ml G418 (geneticin, Gibco, Invitrogen).

### DNA cloning and stable transfection

pCDNA3-luc was generated by inserting the *luciferase *gene from the plasmid pGL3-Basic (Promega) into the *Hind*III-*Xba*I sites of pCDNA3.1 vector (Invitrogen). MC38 cells were grown in 10 cm dishes and were transfected with 4 μg of pCDNA3-luc by the calcium phosphate precipitation method when they reached 50–60% confluence. Post-transfection culture medium was replaced 16 hours later by DMEM 10% FBS supplemented with 400 μg/ml G418. Resistant clones were isolated by individual trypsinization and expanded.

### Bioluminescence imaging

Different clones transfected with the pCDNA3-luc plasmid were plated in 96 well-black plates (Packard) at a density of 100; 500; 1,000; 5,000; 12,500; 25,000; 50,000; 75,000 or 100,000 cells per well for MC38Luc1 cells, or at cell densities above 12,500 cell/well for clones MC38Luc4 and MC38Luc8. Twenty-four hours later, cells were washed in PBS and D-luciferin substrate (Promega) was added at a final concentration of 150 μg/ml. Light emission from culture plates was detected immediately using the IVIS CCD camera system (Xenogen) and analyzed with the Living Image 2.20 software package (Xenogen). For *in vivo *imaging and quantification of light emission, mice were anesthetized with a mixture of Xylacine and Ketamine and 150 mg/kg D-luciferin (100 μl of a 30.3 mg/ml solution dissolved in phosphate-buffered saline) were injected intraperitoneally. Ten minutes later, animals were placed in the dark chamber for light acquisition. Typically, a circular region of interest measuring 3 cm in diameter was defined in the abdomen of mice, and quantification of light emission was performed in photons/second. Time exposure ranged from 1 second to 5 minutes depending on light intensity. A maximum of 10 animals were analyzed at a time.

### Determination of luciferase activity in solution

Fifty to 150 mg of frozen tumor samples were lysed in Reporter Lysis Buffer (Promega) and centrifuged at 13.000 rpm in a cooled microfuge for 10 minutes. The luciferase activity was measured in the supernatant using the Luciferase Reporting Assay System (Promega) in a Berthold microplate luminometer. The luciferase Relative Lights Units (RLUs) obtained for each sample were multiplied by the total tumor weight to obtain an estimation of the RLU/tumor.

### RNA and genomic DNA isolation and qRT-PCRs

Genomic DNA and total RNA was extracted from 50–100 mg frozen tumor samples using QIAmp DNA mini kit only (Qiagen) or TRI reagent (Sigma), respectively, following manufacturer's instructions. Three μg of RNA were treated with DNase I and retro transcribed to cDNA with M-MLV RT in the presence of RNase out reagent (all from Invitrogen). For real time PCR reactions, 2 μl of genomic DNA or cDNA were mixed with specific primers using iQ SYBR Green Supermix (Bio-Rad). Glyceraldehyde-3-phosphate dehydrogenase (GAPDH) was used to normalize gene expression and murine albumin was used to normalize genomic DNA content. Gene expression (specific mRNA content) is represented by the formula 2^ΔCt ^where ΔCt indicates the difference in the threshold cycle between GAPDH and luciferase. Plasmid copy number refers to the copies of the neo resistance gene relative to the copies of the endogenous albumin gene. Standard curves were used to calculate copy numbers in each case. The name and sequence of primers used is as follows: GAPDH forward CCAAGGTCATCCATGACAAC; reverse TGTCATACCAGGAAATGAGC. Albumin forward GATGCTGCTCTTTGGCTATGA; reverse CAGCAGTCAGCCAGTTCACC Neo forward AGATGGATTGCACGCAGGT; reverse TTGCATCAGCCATGATGGA. Firefly Luciferase forward AGAGATACGCCCTGGTTC; reverse ATAAATAACGCGCCCAACAC.

### Immunohistochemistry

MC38 and MC38Luc1 cells were cultured in glass coverslides and fixed in pre-chilled acetone for 4 min. Samples were kept at -20°C before being analyzed. For luciferase protein detection, coverslides were air-dried and washed tree times in phosphate-buffered saline (PBS). Endogenous peroxidase was quenched with Peroxidase Blocking Reagent (DAKO) for 15 min at room temperature. Samples were then washed and incubated with 5 μg/ml of the primary goat anti-luciferase antibody (Cortex) for 1 h at room temperature. After additional washing, samples were incubated with the polyclonal biotinylated anti-goat antibody diluted 1:600 in PBS for 45 min and then were incubated for 45 min with HRP-streptavidin (Amersham). The peroxidase activity was revealed using DAB Substrate Chromogen System (DAKO).

### Mice and tumor cell inoculation

Five to 8 weeks old C57BL/6J female mice were purchased from Harlan (Barcelona, Spain) and were kept in the animal facility at least one week before starting the experiments. Tumors were established by subcutaneous (s.c.) or intrahepatic (i.h.) implantation of cells. For s.c. tumor formation, a total of 10^6 ^cells were injected in the right hind flank. For liver metastases establishment, 5 × 10^5 ^cells were injected into the left liver lobe of mice following medial laparotomy in isofluorane-anesthesized animals. In both cases, cells were resuspended in a total volume of 50 μl saline solution. Tumor size was monitored at indicated time points measuring two perpendicular tumor diameters using a precision calliper. Tumor volume was calculated using the following formula: V = length × width^2 ^× 0.5. In the case of intrahepatic tumors, laparotomies were performed in a subset of the animals at weekly intervals in order to estimate the average tumor volume of the group by direct calliper measurement. Individual animals underwent a maximum of two exploratory laparotomies before they were sacrificed. Survival was checked daily and mice were euthanized if general status was deteriorated. All *in vivo *experiments were performed in accordance with the local animal commission.

### Ultrasonography

Mice were anesthetized with 1.5% isofluorane in oxygen at 0.6 L/min delivered via nose cone and allowed to breathe spontaneously. They were placed in supine position on a feedback-controlled heating pad. Abdomen was shaved, and pre-warmed ultrasound gel was applied on the skin of mice. Recordings were made under continuous ECG monitoring by fixing the electrodes on the limbs. Two-dimension mode U.S imaging was performed by using a dedicated small-animal high resolution imaging unit (VEVO 770; Visualsonics, Toronto, Canada) and a Visualsonics RMV 700-series scanhead with a 30–40 MHz high frequency linear transducer (depth of field 1.5–2.2 mm, field of view-max 14.5–16.5 mm). Tumor diameter measurements were made from digital images captured on cineloops at the time of the study using the software incorporated in the VEVO 770 device. Tumor volumes were calculated as described in the previous section.

### Treatment of colorectal liver metastases by adenoviral-directed expression of IL-12

The High-Capacity (*gutless*) adenoviral vector GL-Ad/RUmIL-12 carrying a liver-specific Mifepristone-inducible system for the expression of murine IL-12 has been previously described [11]. MC38Luc1 cells were inoculated in the liver of C57BL/6 mice, and 3 days later 2.5 × 10^8 ^iu of the virus were injected intravenously dissolved in 200 μl saline solution. One week later, expression of IL-12 was activated by daily intraperitoneal injection of 250 μg/kg Mifepristone for 10 days. Quantification of IL-12 concentration in the serum of animals was performed by ELISA 10 hours after the first induction. Tumor progression was monitored by BLI or direct tumor measurement following laparotomy or necropsy.

### Statistical analysis

We used GraphPad Prism software for statistical analysis. Two-tailed unpaired t-test was used to compare groups of values when n>10. For smaller groups, Mann-Whitney non-parametric test was used.

## Competing interests

The authors declare that they have no competing interests.

## Authors' contributions

MZ carried out the transfection of MC38 cells, the selection and characterization of the MC38Luc1 clone *in vitro*, part of the *in vivo *experiments, the PCR analysis of samples and collaborated in the writing of the manuscript. PA carried out part of the *in vivo *experiments and tissue processing. CB carried out the ultrasonography. JC carried out the production of the GL-Ad/RUmIL-12 vector. GGA coordinated vector production and characterization. COS participated in the design of the study. MGA contributed to the characterization of the MC38Luc1 cells. MGK participated in the design of the study. JP participated in the coordination of the study. RHA conceived the study, participated in the experimental design, participated in the general coordination and wrote the manuscript. All authors read and approved the final manuscript.
